# Synthesis of Ginkgolic Acid Analogues and Evaluation of Their Molluscicidal Activity

**DOI:** 10.3390/molecules16054059

**Published:** 2011-05-17

**Authors:** Peng Zhang, Jiahu Pan, Wanxing Duan, Xuedong Li, Yao Zhang, Yibiao Zhou, Qingwu Jiang, Zuohua Mao, Peizhong Yu

**Affiliations:** 1 School of Pharmacy, Fudan University, Shanghai 201203, China; 2 Shanghai Medical College, Fudan University, Shanghai 200032, China; 3 School of Public Health, Fudan University, Shanghai 200032, China

**Keywords:** ginkgolic acid analogues, synthesis, molluscicidal activity, snail, schistosomiasis

## Abstract

Based on the molluscicidal activity of ginkgolic acids (GAs) isolated from *Ginkgo biloba* L, a series of *Z*/*E* isomers of GA analogues were prepared and evaluated for their molluscicidal activities against the host snail *Oncomelania hupensis*. The results and analysis of the structure-activity relationship revealed that the *E*-isomers showed better molluscicidal activities than their *Z*-isomers. Molluscicidal activities decreased with the shortening of the alkenyl chain lengths.

## 1. Introduction 

Schistosomiasis ranks as the second most prevalent parasitic disease in tropical and subtropical regions of the World after malaria. It is estimated that more than 200 million people are infected and 600 million at risk of infection in approximately 74 countries [1]. China is currently facing a new challenge because this disease has re-emerged in some regions due to an increase in habitats suitable for the snails, these including increased flood areas and large water resource development projects [2]. The snail *Oncomelania hupensis* is the main host responsible for the transmission of this disease in China. 

The use of molluscicides is still considered the most important means to control the transmission of schistosoma parasites by host snails. Although the molluscicide niclosamide (Nic) is the only commercially available molluscicide recommended by the WHO [3], its use is hampered by its high cost and toxicity to fish. This factor has stimulated new avenues of research to identify novel molluscidal agents from natural sources [4,5,6,7,8] and then to improve the molluscicidal properties of known natural compounds, leading to the synthesis of new chemicals analogues with increased activities [9,10,11,12]. 

We have previously described the molluscicidal activities of ginkgolic acids (GAs), a group of 6-alkylsalicylic acids, extracted from the sarcotesta and leaves of *Ginkgo biloba* [13,14]. Five GA monomers were identified and all differed in their chain lengths (13~17 carbon atoms). The alkyl chain was also found to contain different numbers (0~2) of carbon double bonds. The order of molluscicidal activity for these five monomers were C_13:0_ ~C_15:1_ > C_15:0_ > C_17:1_ > C_17:2_. The LD_50_ values after 24 and 48 hours were 20.79 mg∙L^−1^ and 5.06 mg∙L^−1^ for C_13:0_, 27.28 mg∙L^−1^ and 3.27 mg∙L^−1^ for C_15:1_, respectively [14]. Besides the GAs found in the sarcotesta and leaves of *Ginkgo biloba*, additional 6-alkylsalicylic acid derivatives have also been found in other plant species, e.g. anacardic acids isolated from the cashew (*Anacardium occidentale*). They have similar structures to GA C_15:1_ but have a different number (1~3) of double bonds in the alkyl chain. Kubo and colleagues found these compounds to have molluscicidal activities against the South American snail *Biomphalaria glabratus* with LD_50_ values of 1 mg∙L^−1^ for C_15:1_, 0.6 mg∙L^−1^ for C_15:2_, 0.3 mg∙L^−1^ for C_15:3_, respectively, proving that the double bond is related to their activity [15].

All of the known GAs and anacardic acids are derivatives of salicylic acid each having a long carbon side chain substituted at the 6-position. It is suggested that the alkyl group side chain plays an important role in their molluscicidal activities. The major differences in these compounds are the carbon side chain lengths and the number of unsaturated bonds present. No naturally occuring GAs or anacardic acids having side chain length less than 13 carbons in length have been identified. 

In order to understand the structure-activity relationship for these compounds and improve their molluscicidal activity, in this study a series of *Z*/*E* isomers of GA analogues with different chain lengths (5~13 carbon atoms) and phenyl rings based on one added inside double bond have been synthesized from 6-methylsalicylic acid. Their molluscicidal effects against the host snail *Oncomelania hupensis* were evaluated, and the structure-activity relationships were explored.

## 2. Results and Discussion

### 2.1. Synthesis of the compounds

**Scheme 1:**
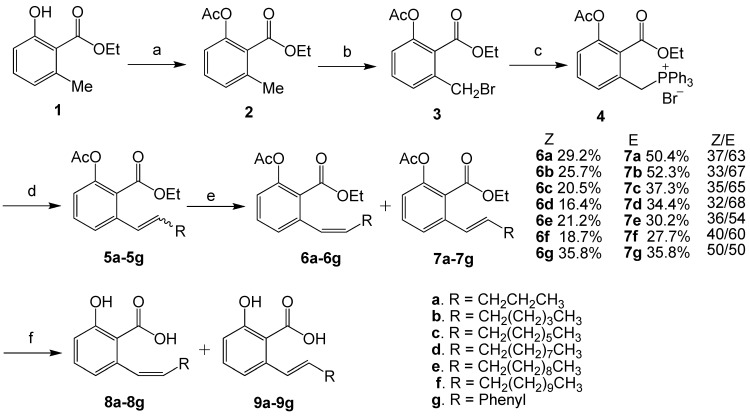
Synthesis of GA analogues from ethyl 6-methylsalicylate.

The unhydrolyzed mixtures of *Z* and *E* isomers **5a–5g** were synthesized from ethyl 6-methylsalicylate according to the reaction sequence delineated in Scheme 1 [16]. These were separated by column chromatography on silica gel. Target compounds **8a**–**8g**, **9a**–**9g** were obtained by ester hydrolysis and extracted from acidic solution with chloroform. Based on the spin–spin coupling constants of the double bond protons, the isomer with smaller coupling constant (*J* = 11~12 Hz) was assigned to the *Z*-configuration, and the larger coupling constant (*J* = 15~16 Hz) was assigned to the *E* configuration. 

### 2.2. Assay for molluscicidal activity

The results of the molluscicidal assays and the representative LD_10_, LD_50_, and LD_90_ values with the confidence limit are shown in Table 1. The above results revealed that all of the *E*-isomers of the synthesized GA analogues had better molluscicidal activities than their *Z*-isomers. According to the LD_50_ values **9f(E)** and **9d(E)** showed the strongest activities among all of the analogues tested. Indeed, **9f** and **9d** were found to have greater activity than that of GA C_13:0_, the most active ingredient among the natural GAs, proving that the molluscicidal activity was improved by the simple addition of an *E* double bond in the alkyl chain. Molluscicidal activities were also found to decrease with the shortening of the alkyl chain lengths in the following sequence: **f** > **d** > **c** > **b** > **a**. The respective LD_50_ values for these compounds were 22.3 μM, 23.6 μM, 27.5 μM, 54.2 μM, and 56.1 μM, respectively. The molluscicidal activity was dramatically decreased when the number of carbon atoms was less than 7. The results of the LD_10_ and LD_90_ data also showed a similar pattern to that of the LD_50_. This pattern of molluscicidal activity were similar to the rules revealed by de Villiers for the varying length acyl chain of one or disubstituted amides [17].

## 3. Experimental 

### 3.1. General 

Melting points were determined on an X4 micro-melting point apparatus, and are uncorrected. IR spectra (KBr) were obtained on Avatar 360 ESP FT-IR spectrometer (*v* in cm^–1^). ^1^H-NMR spectra were recorded in acetone-*d*_6_ or CDCl_3_ on a Varian Mercury Plus 400 NMR spectrometer or a Bruker Avance III 400 MHz NMR spectrometer. The chemical shifts (δ) were given in ppm and the coupling constants (*J*) in Hz. MS spectra were measured with a Thermo-Finnigan model LCQ Classic LC/MS/MS ion trap mass spectrometer and Agilent 1100 chromatography system coupled with an Agilent G1946D mass detector. High-resolution electrospray ionization mass spectra (HRESIMS) were measured on a Bruker Daltonics micrOTOF-QII mass spectrometer. Column chromatography (CC) was performed with silica gel 200–300 mesh (Qingdao Haiyang Chemical Co., Ltd.) and C_18_ reversed-phase silica gel 15–35 μm (Unicorn). Different aldehydes were purchased from Shanghai Jingchun Reagent Co., Ltd. Other reagents were purchased from commercial suppliers and used as received, unless otherwise noted.

### 3.2. Synthesis

#### 3.2.1. Ethyl 6-methylsalicylate (**1**)

Crude **1** was synthesized according to Hamada as a brown oil [18]. The pure colorless crystals of ethyl 6-methylsahcylate were obtained by steam distillation; Mp: 43–44 °C; ^1^H-NMR (CDCl_3_, 400 MHz) δ1.43 (t, *J* = 7.0 Hz, 3H), 2.55 (s, 3H), 4.43 (q, *J* = 7.0 Hz, 2H), 6.70 (d, *J* = 7.4 Hz, 1H), 6.83 (d, *J* = 8.2 Hz, 1H), 7.26 (dd, *J* = 8.2, 7.4 Hz, 1H), 11.38 (s, 1H); ESIMS (positive) *m/z*: 181.1 ([M+H]^+^).

#### 3.2.2. Ethyl O-acetyl-6-methylsalicylate (**2**)

A mixture of acetic anhydride (18 mL) and 6-methylsalicylate (7.2 g, 40 mmol), was stirred at room temperature until the solid dissolved completely. Sulfuric acid (18 drops) was then added dropwise. After 10 min, the mixture was placed into an oil bath of 70 °C for 15 min and then left to cool to room temperature. The reaction mixture was then poured into ice-cold water (50 mL), extracted three times with chloroform (50 mL), and the organic layer was washed with saturated sodium bicarbonate solution several times until no bubble emerges. The sample was then dried over Na_2_SO_4_ to give the desired product **2** as a yellow oil (8.46 g, 95%) [19]. ^1^H-NMR (CDCl_3_, 400 MHz) δ 1.37 (t, *J* = 7.1 Hz, 3H), 2.04 (s, 3H), 2.41 (s, 3H), 4.37 (q, *J* = 7.1 Hz, 2H), 6.96 (d, *J* = 8.3 Hz, 1H), 7.10 (d, *J* = 7.5 Hz, 1H), 7.32 (dd, *J* = 8.3, 7.5 Hz, 1H).

#### 3.2.3. Ethyl O-acetyl-6-bromomethylsalicylate (**3**)

To a stirred solution of compound **2** (8.34 g, 37.6 mmol) dissolved in carbon tetrachloride (60 mL) were added a mixture of N-bromosuccinimide (7.44 g, 41.8 mmol) and azobisisobutyronitrile (246 mg, 0.15 mmol). This solution was then heated at reflux for 3 h. After cooling, the reaction mixture was filtered and the filtrate was condensed under reduced pressure to give the crude product Chromatographic purification of the crude material (silica gel, petroleum ether-EtOAc = 15:1) gave the bromide **3** (10.24 g, 90%) as a white powder. This power was directly used for the next reaction without further identification.

#### 3.2.4. 3-Acetoxy-2-ethoxycarbonylbenzyltriphenylphosphonium bromide (**4**)

A mixture of compound **3** (10.2 g, 34 mmol) and triphenylphosphine (8.91 g, 34 mmol) was added to a magnetically stirred round bottomed flask at 0 °C, and then slowly heated to 130 °C. After all the power melted together, the reaction mixture was cooled to 70 °C. A small amount of chloroform was added and refluxed until all the solid had dissolved. Anhydrous ether (30 mL) was then added and the reaction mixture was filtered and to give the phosphonium salt as a slightly yellow powder (18.7 g, 98%). 

#### 3.2.5. General procedure for preparation of ethyl *O*-acetyl-6-(1-alkenyl)salicylates **5a**–**5g**

The phosphonium salt **4** (563 mg, 1 mmol) was dissolved in DMSO (5 mL). Triethylamine (0.170 mL, 1.2 mmol) and different aldehydes was added. The reaction mixture was heated to 125 °C for 21–26 h, then allowed to cool to ambient temperature, poured onto ice-cold water (25 mL), and extracted three times with ether (each 50 mL). The organic layer was washed with water (50 mL) and saturated brine (50 mL), and dried over Na_2_SO_4_. The solution was then filtered, and concentrated under reduced pressure to give the crude product as a mixture of geometric isomers.

#### 3.2.6. Separation of *Z* and *E* isomers **6a**–**6g** and **7a**–**7g** of ethyl O-acetyl-6-(1-alkenyl)salicylates

Chromatographic separation of the crude material (silica gel, *n*-hexane and acetone, about 30:1) gave the Z (**6a–6g**) and E (**7a–7g**) isomers as yellow oils with a ratio of 37/63 to 50/50 (*Z*/*E*) (Scheme 1).

#### 3.2.7. General procedure for preparation of 6-(1-alkenyl)salicylic acids

Triple molecular quantities of 1N NaOH and the ester **6a–6g** and **7a**–**7g**, in EtOH was heated at reflux for 5–8 h, respectively. After removal of the solvent, the residue was acidified to pH 2 with 10% HCl, diluted with water, and extracted three times with dichloromethane. The organic layer was washed with water and saturated brine, dried over Na_2_SO_4_, and concentrated *in vacuo* to give the crude products. These were purified by chromatography (C_18_, MeOH-H_2_O = 17:3) to obtain the salicylic acids (Z-isomer **8a**–**8g** and E-isomer **9a**–**9g**) as yellowish or white powders.

#### 3.2.8. (*Z*)-6-(1-Pentenyl)salicylic acid (**8a**)

(*Z*)-6-(1-Pentenyl)salicylic acid (**8a**, 49.1 mg, 94%) was obtained as a yellowish powder from the hydrolysis of **6a** (70 mg, 0.25 mmol) by 1N NaOH (0.75 mL, 0.75 mmol) in EtOH (1.54 mL) for 5 h. Mp 85–86 °C; IR (KBr, cm^−1^) *ν* 3425, 3027, 2956, 2870, 1646, 1602, 1486, 1448, 1301, 1237, 1164; ^1^H-NMR (acetone-*d*_6_, 400 MHz) δ 0.85 (t, *J* = 7.4 Hz, 3H), 1.40 (m, 2H), 2.08 (m, 2H), 5.63 (dt, *J* = 11.4, 7.4 Hz, 1H), 6.76 (d, *J* = 7.4 Hz, 1H), 6.85 (d, *J* = 11.4 Hz, 1H), 6.87 (d, *J* = 8.2 Hz, 1H), 7.43 (dd, *J* = 8.2, 7.4 Hz, 1H); HRESIMS found: 205.0854. Calcd: 205.0865 for C_12_H_13_O_3_ ([M-H]^−^).

#### 3.2.9. (*E*)-6-(1-Pentenyl)salicylic acid (**9a**)

(*E*)-6-(1-Pentenyl)salicylic acid (**9a**, 85.4 mg, 88%) was obtained as a yellowish powder from the hydrolysis of **7a** (130 mg, 0.47 mmol) by 1N NaOH (1.41 mL, 1.41 mmol) in EtOH (2.86 mL) for 6 h. Mp 96–98 °C, IR (KBr, cm^−1^) *ν* 3414, 3052, 2958, 2930, 2873, 1652, 1602, 1445, 1245, 1170; ^1^H-NMR (acetone-*d*_6_, 400 MHz) δ 0.95 (t, *J* = 7.2 Hz, 3H), 1.47 (m, 2H), 2.19 (m, 2H), 6.04 (dt, *J* = 15.7, 7.0 Hz, 1H), 6.83 (d, *J* = 8.2 Hz, 1H), 7.01 (d, *J* = 7.8 Hz, 1H), 7.12 (d, *J* = 15.7 Hz, 1H), 7.38 (dd, *J* = 8.2, 7.8 Hz, 1H); HRESIMS found: 205.0807. Calcd: 205.0865 for C_12_H_13_O_3_ ([M-H]^−^).

#### 3.2.10. (*Z*)-6-(1-Heptenyl)salicylic acid (**8b**)

(*Z*)-6-(1-Heptenyl)salicylic acid (**8b**, 46.5 mg, 93%) was obtained as a yellowish powder from the hydrolysis of **6b** (65 mg, 0.21 mmol) by 1N NaOH (0.63 mL, 0.63 mmol) in EtOH (1.43 mL) for 5 h. Mp 55–56 °C, IR (KBr, cm^−1^) *ν* 3423, 3058, 2959, 2925, 2844, 1652, 1599, 1455, 1384, 1113; ^1^H-NMR (acetone-*d*_6_, 400 MHz) δ 0.83 (t, *J* = 7.0 Hz, 3H), 1.23 (m, 4H), 1.40 (m, 2H), 2.10 (m, 2H), 5.63 (dt, *J* = 11.7, 7.4 Hz, 1H), 6.76 (d, *J* = 7.4 Hz, 1H), 6.81 (d, *J* = 11.7 Hz, 1H), 6.87 (d, *J* = 8.2 Hz, 1H), 7.43 (dd, *J* = 8.2, 7.4 Hz, 1H); HRESIMS found: 233.1166. Calcd: 233.1178 for C_14_H_17_O_3_ ([M-H]^−^).

#### 3.2.11. (*E*)-6-(1-Heptenyl)salicylic acid (**9b**)

(*E*)-6-(1-Heptenyl)salicylic acid (**9b**, 108.4 mg, 88%) was obtained as a yellowish powder from the hydrolysis of **7b** (160 mg, 0.53 mmol) by 1N NaOH (1.59 mL, 1.59 mmol) in EtOH (3.52 mL) for 6 h. Mp 70–72 °C, IR (KBr, cm^−1^) *ν* 3430, 3035, 2957, 2925, 2851, 1655, 1601, 1446, 1243, 1219, 1171, 1064; ^1^H-NMR (acetone-*d*_6_, 400 MHz) δ 0.87 (m, 3H), 1.35 (m, 4H), 1.49 (m, 2H), 2.21 (m, 2H), 6.04 (dt, *J* = 15.7, 6.7 Hz, 1H), 6.82 (d, *J* = 8.2 Hz, 1H), 7.01 (d, *J* = 7.4 Hz, 1H), 7.11 (d, *J* = 15.7 Hz, 1H), 7.37 (*J* = 8.2, 7.4 Hz, 1H); HRESIMS found: 257.1142. Calcd: 257.1154 for C_14_H_18_NaO_3_ ([M+Na]^+^). 

#### 3.2.12. (*Z*)-6-(1-Nonenyl)salicylic acid (**8c**)

(*Z*)-6-(1-Nonenyl)salicylic acid (**8c**, 44.5 mg, 94%) was obtained as a yellowish powder from the hydrolysis of **6c** (60 mg, 0.18 mmol) by 1N NaOH (0.54 mL, 0.54 mmol) in EtOH (1.32 mL) for 5 h. Mp 40–42 °C; IR (KBr, cm^–1^) *ν* 3421, 3029, 2956, 2924, 2854, 1651, 1601, 1449, 1384, 1298, 1188; ^1^H-NMR (acetone-*d*_6_, 400 MHz) δ 0.84 (t, *J* = 7.0 Hz, 3H), 1.22 (m, 8H), 1.40 (m, 2H), 2.11 (m, 2H), 5.62 (dt, *J* = 11.4, 7.4 Hz, 1H), 6.76 (d, *J* = 7.8 Hz, 1H), 6.82 (d, *J* = 11.4 Hz, 1H), 6.87 (d, *J* = 8.2 Hz, 1H), 7.42 (dd, *J* = 8.2, 7.8 Hz, 1H); HRESIMS found: 261.1433. Calcd: 261.1491 for C_16_H_21_O_3_ ([M-H]^−^).

#### 3.2.13. (*E*)-6-(1-Nonenyl)salicylic acid (**9c**)

(*E*)-6-(1-Nonenyl)salicylic acid (**9c**, 79 mg, 87%) was obtained as a yellowish powder from the hydrolysis of **7c** (115 mg, 0.35 mmol) by 1N NaOH (1.05 mL, 1.05 mmol) in EtOH (2.53 mL) for 7 h. Mp 75–77 °C, IR (KBr, cm^−1^) *ν* 3416, 3058, 2959, 2925, 2850, 1640, 1601, 1440, 1300, 1215, 1171, 707; ^1^H-NMR (acetone-*d*_6_, 400 MHz) δ 0.88 (t, *J* = 7.0 Hz, 3H), 1.22 (m, 8H), 1.49 (m, 2H), 2.22 (m, 2H), 6.05 (dt, *J* = 15.7, 6.7 Hz, 1H), 6.83 (d, *J* = 8.6 Hz, 1H), 7.01 (d, *J* = 7.4 Hz, 1H), 7.13 (d, *J* = 15.7 Hz, 1H), 7.37 (dd, *J* = 8.6, 7.4 Hz, 1H); HRESIMS found: 261.1486. Calcd: 261.1491 for C_16_H_21_O_3_ ([M-H]^−^).

#### 3.2.14. (*Z*)-6-(1-Undecenyl)salicylic acid (**8d**)

(*Z*)-6-(1-Undecenyl)salicylic acid (**8d**, 37.2 mg, 92%) was obtained as a yellowish powder from the hydrolysis of **6d** (50 mg, 0.14 mmol) by 1N NaOH (0.42 mL, 0.42 mmol) in EtOH (1.10 mL) for 5 h. Mp 47–49 °C; IR (KBr, cm^−1^) *ν* 3418, 3046, 2956, 2924, 2853, 1650, 1634, 1586, 1455, 1384, 1206, 1114, 1063; ^1^H-NMR (CDCl_3_, 400 MHz) δ 0.89 (t, *J* = 6.8 Hz, 3H), 1.24 (m, 12H), 1.41 (m, 2H), 2.09 (m, 2H), 5.82 (dt, *J* = 11.2, 7.6 Hz, 1H), 6.75 (d, *J* = 7.6 Hz, 1H), 6.79 (d, *J* = 11.2 Hz, 1H), 6.96 (d, *J* = 8.4 Hz, 1H), 7.25 (dd, *J* = 8.4, 7.6 Hz, 1H); HRESIMS found: 289.1835. Calcd: 289.1804 for C_18_H_25_O_3_ ([M-H]^−^). 

#### 3.2.15. (*E*)-6-(1-Undecenyl)salicylic acid (**9d**)

(*E*)-6-(1-Undecenyl)salicylic acid (**9d**, 73.9 mg, 83%) was obtained as a yellowish powder from the hydrolysis of **7d** (110 mg, 0.31 mmol) by 1N NaOH (0.93 mL, 0.93 mmol) in EtOH (2.42 mL) for 7 h. Mp 79–81 °C; IR (KBr, cm^−1^) *ν* 3444, 3058, 2960, 2922, 2849, 1642, 1600, 1440, 1213, 1171; ^1^H-NMR (acetone-*d*_6_, 400 MHz) δ 0.87 (t, *J* = 6.7 Hz, 3H), 1.29 (m, 12H), 1.48 (m, 2H), 2.22 (m, 2H), 6.04 (dt, *J* = 15.7, 6.7 Hz, 1H), 6.83 (d, *J* = 8.2 Hz, 1H), 7.01 (d, *J* = 7.8 Hz, 1H), 7.12 (d, *J* = 15.7 Hz, 1H), 7.37 (dd, *J* = 8.2, 7.8 Hz, 1H); HRESIMS found: 289.1787. Calcd: 289.1804 for C_18_H_25_O_3_ ([M-H]^−^). 

#### 3.2.16. (*Z*)-6-(1-Dodecenyl)salicylic acid (**8e**)

(*Z*)-6-(1-Dodecenyl)salicylic acid (**8e**, 54.8 mg, 96%) was obtained as a white powder from the hydrolysis of **6e** (70 mg, 0.19 mmol) by 1N NaOH (0.57 mL, 0.57 mmol) in EtOH (1.54 mL) for 5 h. Mp 52–54 °C; IR (KBr, cm^−1^) *ν* 3386, 3058, 2956 ,2917, 2850, 1728, 1659, 1600, 1450, 1383, 1288, 1189; ^1^H-NMR (acetone-d6, 400 MHz) δ 0.85 (t, *J* = 7.0 Hz, 3H), 1.25 (m, 14H), 1.36 (m, 2H), 2.10 (m, 2H), 5.61 (dt, *J* = 11.4, 7.4 Hz, 1H), 6.75 (d, *J* = 7.4 Hz, 1H), 6.81 (d, *J* = 11.4 Hz, 1H), 6.86 (d, *J* = 8.2 Hz, 1H), 7.41 (dd, *J* = 8.2, 7.4 Hz, 1H); HRESIMS found: 303.1927. Calcd: 303.1960 for C_19_H_27_O_3_ ([M-H]^−^). 

#### 3.2.17. (*E*)-6-(1-Dodecenyl)salicylic acid (**9e**)

(*E*)-6-(1-Dodecenyl)salicylic acid (**9e**, 75 mg, 88%) was obtained as a white powder from the hydrolysis of **7e** (105 mg, 0.28 mmol) by 1N NaOH (0.84 mL, 0.84 mmol) in EtOH (2.31 mL) for 8 h. Mp 82–84 °C; IR (KBr, cm^−1^) *ν* 3446, 3058, 2959, 2922, 2850, 1641, 1601, 1469, 1443, 1302, 1216, 1170; ^1^H-NMR (acetone-*d*_6_, 400 MHz) δ 0.87 (t, *J* = 7.0 Hz, 3H), 1.28 (m, 14H), 1.48 (m, 2H), 2.22 (m, 2H), 6.04 (dt, *J* = 15.7, 6.7 Hz, 1H), 6.83 (d, *J* = 8.2 Hz, 1H), 7.01 (d, *J* = 7.4 Hz, 1H), 7.12 (d, *J* = 15.7 Hz, 1H), 7.37 (dd, *J* = 8.2, 7.4 Hz, 1H); HRESIMS found: 303.1966. Calcd: 303.1960 for C_19_H_27_O_3_ ([M-H]^−^). 

#### 3.2.18. (*Z*)-6-(1-Tridecenyl)salicylic acid (**8f**)

(*Z*)-6-(1-Tridecenyl)salicylic acid (**8f**, 47.3 mg, 89%) was obtained as a white powder from the hydrolysis of **6f** (65 mg, 0.17 mmol) by 1N NaOH (0.51 mL, 0.51 mmol) in EtOH (1.43 mL) for 5 h. Mp 55–57 °C; IR (KBr, cm^−1^) *ν* 3431, 3055, 2957, 2921, 2850, 1731, 1656, 1600, 1448, 1384, 1300, 1189; ^1^H-NMR (acetone-*d*_6_, 400 MHz) δ 0.87 (t, *J* = 6.7 Hz, 3H), 1.25 (m, 16H), 1.37 (m, 2H), 2.11 (m, 2H), 5.62 (dt, *J* = 11.3, 7.4 Hz, 1H), 6.76 (d, *J* = 7.4 Hz, 1H), 6.83 (d, *J* = 11.3 Hz, 1H), 6.87 (d, *J* = 8.2 Hz, 1H), 7.4 (dd, *J* = 8.2, 7.4 Hz, 1H); HRESIMS found: 317.2152. Calcd: 317.2117 for C_20_H_29_O_3_ ([M-H]^−^). 

#### 3.2.19. (*E*)-6-(1-Tridecenyl)salicylic acid (**9f**)

(*E*)-6-(1-Tridecenyl)salicylic acid (**9f**, 73.8 mg, 90%) was obtained as a white powder from the hydrolysis of **7f** (100 mg, 0.26 mmol) by 1N NaOH (0.78 mL, 0.78 mmol) in EtOH (2.2 mL) for 8 h. Mp 90–92 °C; IR (KBr, cm^−1^) *ν* 3425, 3063, 2961, 2920, 2848, 1642, 1601, 1467, 1439, 1304, 1214, 1171; ^1^H-NMR (acetone-*d*_6_, 400 MHz) δ 0.87 (t, *J* = 7.0 Hz, 3H), 1.29 (m, 16H), 1.49 (m, 2H), 2.22 (m, 2H), 6.05 (dt, *J* = 15.7, 7.0 Hz, 1H), 6.83 (d, *J* = 8.2 Hz, 1H), 7.01 (d, *J* = 7.4 Hz,1H), 7.11 (d, *J* = 15.7 Hz, 1H), 7.37 (dd, *J* = 8.2, 7.4 Hz, 1H); HRESIMS found: 317.2114. Calcd: 317.2117 for C_20_H_29_O_3_ ([M-H]^−^). 

#### 3.2.20. (*Z*)-6-Phenylethenyl salicylic acid (**8g**) 

(*Z*)-6-Phenylethylenyl salicylic acid (**8g**, 72 mg, 93%) was obtained as a yellowish powder from the hydrolysis of **6g** (100 mg, 0.32 mmol) by 1N NaOH (0.96 mL, 0.96 mmol) in EtOH (2.2 mL) for 5 h. Mp 84–86 °C; IR (KBr, cm^−1^) *ν* 3422, 3059, 3025, 2948, 2924, 2852, 1733, 1652, 1599, 1448, 1398, 1297, 1205, 1171; ^1^H-NMR (acetone-*d*_6_, 400 MHz) δ 6.57 (d, *J* = 12.1 Hz, 1H), 6.62 (d, *J* = 7.4 Hz, 1H), 6.85 (d, *J* = 8.6 Hz, 1H), 7.05–7.18 (m, 6H), 7.23 (t, *J* = 7.8 Hz, 1H); HRESIMS found: 263.0677. Calcd: 263.0684 for C_15_H_12_NaO_3_ ([M+Na]^+^). 

#### 3.2.21. (*E*)-6-Phenylethenyl salicylic acid (**9g**)

(*E*)-6-Phenylethylenyl salicylic acid (**9g**, 70.8 mg, 92%) was obtained as a yellowish powder from the hydrolysis of **7g** (100 mg, 0.32 mmol) by 1N NaOH (0.96 mL, 0.96 mmol) in EtOH (2.2 mL) for 5 h. Mp 130–132 °C; IR (KBr, cm^−1^) *ν* 3443, 3061, 3025, 2959, 2925, 2851, 1674, 1652, 1600, 1449, 1384, 1205, 1171; ^1^H-NMR (acetone-*d*_6_, 400 MHz) δ 6.88 (d, *J* = 8.2 Hz, 1H), 6.97 (d, *J* = 16.0 Hz, 1H), 7.20 (d, *J* = 7.8 Hz, 1H), 7.27 (m, 1H), 7.34–7.43 (m, 3H), 7.59 (d, *J* = 7.8 Hz, 2H), 8.16 (d, *J* = 16.0 Hz, 1H); HRESIMS found: 239.0736. Calcd: 239.0786 for C_15_H_11_O_3_ ([M-H]^−^). 

### 3.3. Molluscicidal activity tests

The molluscicidal test was carried out in accordance with WHO guidelines for laboratory molluscicidal tests [20]. The *Oncomelania hupensis* snails were collected from the epidemic area of Schistosomiasis in Anhui Province, China. Healthy adult snails were used in all molluscicidal tests. Each compound was dissolved into a small amount of alcohol (less than 0.1% final concentration) and then diluted with dechlorinated water. Niclosamide (Nic) was used as a positive control and dechlorinated water as the control. The molluscicidal test was carried out using the immersion method. Snails (20 snails in each group) were placed into beakers containing each test compound at different concentrations for a period of 48 h at 22 ± 2 °C. Each beaker was covered with nylon netting to prevent the snails from escaping. Snail mortality was carefully checked and all tests were carried out in duplicate. The LD_10_, LD_50_ and LD_90_ values were determined using Probit analysis [21] of the mortality data.

## 4. Conclusions

Synthetic GA analogues represent a new class of chemical entities with promising molluscicidal activities. In this study, a series of *Z*/*E* isomers were prepared and evaluated for their molluscicidal activities against the host snail *O. hupensis.* The results and analysis of the structural–activity relationship revealed that the *E*-isomers were better molluscicidal agents than their respective *Z*-isomers. Activity was found to decrease regularly with the shortening of the alkyl chain lengths. New lead compounds have been identified that warrant further study.

## Figures and Tables

*Reagents and conditions*: (a) (CH_3_CO)_2_O, H_2_SO_4_, 70 °C, 15min, 95%; (b) CCl_4_, NBS, AIBN, 90%; (c) Ph_3_P, 0 to 130 °C, CHCl_3_, (CH_3_CH_2_)_2_O, 98%; (d) R-CHO, Et_3_N, DMSO, 125 °C, 21~26 h, 46.4~80%; (e) separated by silica gel column chromatography with gradient mixtures of *n*-hexane-acetone (30:1); (f) EtOH, NaOH, reflux, 5~8 h, 83~96%.

**Table 1:** Molluscicidal activity (μM) of the Z/E isomers of GA analogues against snail *O.hupensis* under laboratory conditions and 48 h exposure.

Compound	**LD_10_ (C.L.)^a^**		**LD_50_ (C.L.)^a^**		**LD_90_ (C.L.)^a^**	
	**8** (Z)	**9** (E)	**8** (Z)	**9** (E)	**8** (Z)	**9** (E)
**a**	27.2 (9.3–44.1)	14.6 (7.6–57.8)	107.2 (32.4–133.8)	56.1 (16.7–122.6)	786.6 (322.6–1213.8)	115.9 (35.1–643.1)
**b**	33.6 (7.5–75.6)	22 (6.9–35.3)	73.1 (48.4–130.4)	54.2 (21.4–132.2)	243.6 (134.9–1215)	214.5 (78.3–462.8)
**c**	12.5 (4.8–19.6)	6.5 (0.8–9.1)	50.9 (6.5–68.7)	27.5 (14.8–87.1)	98.2 (25.5–150.6)	74.3 (34.2–95.0)
**d**	18.4 (1.7–27.7)	10.9 (1.6–17.3)	43.7 (22.9–89.2)	23.6 (10.5–26.9)	135.7 (14.2–199.3)	68.3 (63.4–801.6)
**e**	11.1 (3.0–17.0)	7.7 (0.3–15.0)	56.9 (24.9–63.6)	31.4 (21.1–80.3)	362.9 (83.4–595.5)	88.7 (46.7–121.1)
**f**	13.3 (0.3–26.6)	5.5 (1.5–6.2)	47.2 (31.4–57.7)	22.4 (4.3–25.3)	338.7 (82.2–364.6)	50.9 (23.3–62.8)
**g**	14.6 (3.3–25.9)	11.4 (3.6–18.8)	64.4 (40.1–127.7)	37.2 (23.9–57.0)	283.9 (138.6–2001)	122.0 (74.6–366.6)
GA C_13:0_ *	1.6 (0.8–8.6)		29.2 (4.2–60.8)		64.6 (37.7–180.2)	
Niclosamide	0.7 (0.3–0.9)		1.5 (1.1–2.1)		3.4 (2.3–7.9)	

The above data were the average from 4 dependent experiments; * GA C_13:0_ was isolated from the sarcotesta and leave of *Ginkgo biloba* L.; **^a^** Confidence limit.
